# The Effect of Pharmacist-Led Intervention on Surgical Antibacterial Prophylaxis (SAP) at an Orthopedic Unit

**DOI:** 10.3390/antibiotics10121509

**Published:** 2021-12-09

**Authors:** Adina Fésüs, Ria Benkő, Mária Matuz, Orsolya Kungler-Gorácz, Márton Á. Fésüs, Tamás Bazsó, Zoltán Csernátony, Gábor Kardos

**Affiliations:** 1 Central Clinical Pharmacy, Clinical Center, University of Debrecen, H-4032 Debrecen, Hungary; fesus.adina@pharm.unideb.hu (A.F.); goracz.orsolya@med.unideb.hu (O.K.-G.); 2 Department of Pharmacodynamics, Faculty of Pharmacy, University of Debrecen, H-4032 Debrecen, Hungary; 3 Doctoral School of Pharmaceutical Sciences, University of Debrecen, H-4032 Debrecen, Hungary; 4 Clinical Pharmacy Department, Faculty of Pharmacy, University of Szeged, H-6725 Szeged, Hungary; benko.ria@med.u-szeged.hu (R.B.); matuz.maria@szte.hu (M.M.); 5 Central Pharmacy, Albert Szent Györgyi Medical Center, University of Szeged, H-6725 Szeged, Hungary; 6 Department of Emergency Medicine, Albert Szent Györgyi Medical Center, University of Szeged, H-6725 Szeged, Hungary; 7 Department of Metagenomics, University of Debrecen, H-4032 Debrecen, Hungary; 8 Department of Traumatology and Hand Surgery, Faculty of Medicine, University of Debrecen, H-4031 Debrecen, Hungary; dr.fesus.marton@kenezy.unideb.hu; 9 Department of Orthopedic Surgery, Faculty of Medicine, University of Debrecen, H-4032 Debrecen, Hungary; bazso.tamas@med.unideb.hu (T.B.); csz@med.unideb.hu (Z.C.)

**Keywords:** antibiotic stewardship consultation, surgical antibacterial prophylaxis, total hip arthroplasty, total knee arthroplasty, cost of surgical antibacterial prophylaxis, antibiotic exposure

## Abstract

Perioperative antibiotic use is a common reason for antibiotic misuse. Evidence suggests that adherence to SAP guidelines may improve outcomes. The purpose of this study was to analyze the impact of pharmacist-led antibiotic stewardship interventions on SAP guideline compliance. The study was conducted at an Orthopedic Department of a tertiary care medical center. SAP compliance and antibiotic exposure in the pre-intervention and intervention period was compared using chi-square, Fisher exact, and Mann-Whitney tests, as appropriate. Prophylactic antibiotic use in orthopedic joint arthroplasties (overall guideline adherence: agent, dose, frequency, duration), clinical outcomes (length of stay-LOS, number of surgical site infections-SSIs), antibiotic exposure and direct antibiotic costs were compared between pre-intervention and intervention periods. Significant improvement in mean SAP duration (by 42.9%, 4.08 ± 2.08 vs. 2.08 ± 1.90 days, *p* ˂ 0.001), and overall guideline adherence regarding antibiotic use (by 56.2%, from 2% to 58.2%, *p* ˂ 0.001) were observed. A significant decrease was observed in antibiotic exposure in SAP (by 41%, from 6.07 ± 0.05 to 3.58 ± 4.33 DDD/patient, *p* ˂ 0.001), average prophylactic antibiotic cost (by 54.8%, 9278.79 ± 6094.29 vs. 3598.16 ± 3354.55 HUF/patient), and mean LOS (by 37.2%, from 11.22 ± 6.96 to 7.62 ± 3.02 days, *p* < 0.001); and a slight decrease in the number of confirmed SSIs was found between the two periods (by 1.8%, from 3% to 1.2%, *p* = 0.21). Continuous presence of the clinical pharmacist led to significant improvement in SAP guideline adherence, which was accompanied by decreased antibiotic exposure and cost.

## 1. Introduction

Joint arthroplasties are frequently performed life-enhancing procedures. The need for these surgical procedures continues to rise. Although arthroplasties belong to clean surgical procedures [1,2], surgical site infections (SSIs) are not uncommon. SSIs are defined as infections occurring after arthroplasties, and can involve superficial or deep tissues at the operation site. Furthermore, SSIs may result in periprosthetic joint infection (PJI). PJI is defined as infection involving the implants and adjacent tissues, which is one of the most threatening complications in orthopedic arthroplasties, being responsible for excess morbidity and increased costs [3,4]. Systemic antibacterial prophylaxis is a standard practice to prevent SSI, especially where implants are used [5,6]. Inadequate Surgical Antibacterial Prophylaxis (SAP) can significantly increase antibiotic consumption in surgical wards. Inappropriate prophylaxis includes choosing an inappropriate drug, underdosing, as well as inadequate timing or prolonged administration, which also subsequently contribute to the development of antibiotic resistance [7]. Adherence to the Clinical Practice Guidelines for Antimicrobial Prophylaxis in Surgery recommended by ASHP (American Society of Hospital Pharmacists) may be greatly enhanced by providing counselling for prescribers via consultation with pharmacists [8,9], who can play a key role in controlling inappropriate prophylactic antibiotic usage and correcting suboptimal drug regimens. Based on international studies the involvement of the pharmacist in SAP has led to a number of positive outcomes [7,10,11]. To date, there are no published reports from Hungary regarding the role of pharmacists in optimizing and promoting rational use of antibiotics in SAP.

The purpose of this study was to analyze the impact of pharmacist-led antibiotic stewardship interventions on compliance with SAP among patients undergoing joint arthroplasties, as well as antibiotic exposure and cost in SAP.

## 2. Results

**Figure 1:**
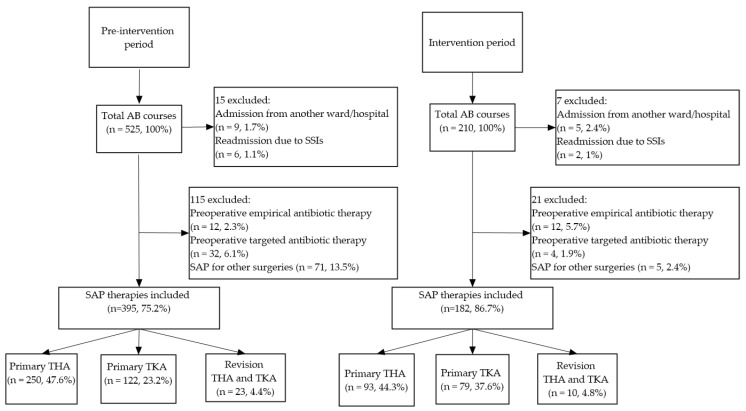
Patients included and excluded from the study. AB-antibiotic, SAP-Surgical Antibacterial Prophylaxis, THA-Total Hip Arthroplasty, TKA-Total Knee Arthroplasty.

In the pre-intervention (12 months) and the intervention period (7 months) data of 525 and 210 patients, respectively, were collected. Of them, 130 patients were excluded in the pre-intervention period and 28 patients in the intervention period, due to various reasons (see Figure 1).

### 2.1. Patient’s Characteristics

The characteristics of patients and surgical procedures are described in Table 1. Although the number of patients differed, no significant differences were found regarding their age, gender, median body weight, and diagnosis for primary arthroplasty between the two periods. In both periods almost two thirds of the patients were female, and the number of THAs was also higher than the number of TKAs (Table 1).

### 2.2. SAP Characteristics and Pharmacist intervention

#### 2.2.1. Agent Selection, Dosage

The characteristics of SAP and outcomes are summarized in Table 2. Cefuroxime was used for prophylaxis in both periods in the vast majority of arthroplasties (88.1% vs. 87.9%). Ciprofloxacin was and remained the most frequently used non-recommended agent (5.6% vs. 7.1%), even in beta-lactam allergy, when vancomycin or clindamycin were recommended by guidelines. No significant changes in the use of guideline non-adherent combinations with metronidazole (5% vs. 2.2%, *p* > 0.05) were observed between the two periods. However, the use of the guideline adherent combination of cefuroxime and amikacin slightly increased (from 0.5% to 2.2%, *p* > 0.05) in the intervention period. Data show no differences in dose and frequency of antibiotics used for SAP (Table 2).

#### 2.2.2. Timing and Duration of SAP

No data on the timing of the first dose of SAP were collected. Considering all arthroplasties together, we found a significant difference in the mean duration of SAP between the two periods (42.9%, pre-intervention: 4.08 ± 2.08 vs. intervention: 2.42 ± 1.90 days, *p* ˂ 0.001). At the same time, guideline adherence in terms of SAP duration improved significantly (by 59.3%, from 5% to 64.3%, *p* ˂ 0.001) (Table 2). These rates are more pronounced for primary arthroplasties (both THA and TKA), where guideline adherent one-day SAP increased significantly (by 59%, from 2% to 61%, *p* ˂ 0.001).

#### 2.2.3. Antibiotic Exposure and Cost in SAP

Antibiotic exposure in SAP decreased significantly (by 41%, from 6.07 ± 0.05 to 3.58 ± 4.33 DDD/patient, *p* ˂ 0.001) (Table 2). When analyzing primary arthroplasties, decreases were also found to be significant (5.57 ± 2.83 vs. 3.03 ± 2.92 DDD/person, *p* < 0.001). As expected, the decrease of SAP duration led to significantly lower prophylaxis costs after intervention (by 54.8%, 9278.79 ± 6094.29 vs. 3598.16 ± 3354.55 HUF/patient, *p* < 0.001) (Table 2). Overall cost was reduced 2.2-fold (*p* < 0.001).

#### 2.2.4. Clinical Outcomes: LOS (Length of Stay) and SSIs

Comparing the pre-intervention and intervention periods, overall LOS decreased significantly for all types of arthroplasties (by 37.2%, from 11.22 ± 6.96 to 7.62 ± 3.02 days, respectively, *p* < 0.001) (Table 2). Hospital readmissions due to SSI were followed for 60 days after discharge in both periods. The mean time of the diagnosis of SSIs was eight days (range 1–23, *p* ≥ 0.05) (Table 2). A slight decrease (1.8%, from 3% to 1.2%, *p* = 0.21) in the number of confirmed SSIs was found between the two periods. The number of suspected SSIs was higher (11.6% vs. 2.3%, *p* ˂ 0.001), which is consistent with the fact that empirical or targeted administration of antibiotics was deemed necessary in 19.5% of the pre-intervention cases and 2.7% of the cases in the intervention period (*p* < 0.001) (Table 2).

## 3. Discussion

Antibiotic resistance occurs everywhere in the world [12]. The antimicrobial stewardship program (ASP) aims to slow the emergence of antibiotic resistance [13]. However, currently, there is no official national ASP strategy in Hungary. Different healthcare providers have their own strategies, in which the opportunities and responsibilities of the clinical pharmacists are defined based on the WHO document [14]. Optimizing SAP is an important part of ASP in surgical departments.

Even though SAP is of major importance in the prevention of SSIs [15,16], it is still a hotbed for antibiotic overuse and misuse. However, pharmacist intervention can have a significant impact on SAP compliance in orthopedic surgical procedures, particularly on the overall guideline adherence that may result in significant decrease in antibiotic exposure, cost, and clinical outcomes.

In the present study, we found that in the pre-intervention period irrational antibiotic use was quite frequent for SAP in orthopedic surgery. Redundant antibiotic combinations of beta-lactams or quinolones with metronidazole, and prolonged SAP duration contributed to the high total antibiotic consumption and costs of SAP. In the intervention period we implemented daily pharmacist intervention, which resulted in remarkable changes in several parameters of SAP and clinical outcomes (Table 2). There are several studies where pharmacist intervention was shown to be particularly effective in increasing the guideline adherence rates of SAP in terms of agent selection, dosing, timing, and duration [17,18,19]. Based on the available evidence, it was found that clinical pharmacists play an important role in all aspects of rational antibiotic use; however, this recognition is still in its early stages in orthopedics [20], which highly limits our comparison with similar studies.

### 3.1. Overall Guideline Adherence

Several studies assessing compliance with SAP guidelines have been published. In the literature, the most important types of non-adherence to guidelines are (i) starting prophylaxis before the day of surgery [21,22], (ii) prolongation of prophylaxis in the postoperative period [22], and (iii) unnecessary use of broad-spectrum agents [22]. In the present study, prophylaxis prior to the day of surgery was considered preoperative empirical antibiotic therapy; therefore, patients receiving such therapies were excluded. Regarding the timing of prophylaxis, although the optimal and recommended timing is 30 min to one hour prior to incision, and antibiotics should consequently be administered in the operation room, the time of their use was generally found to receive low priority among anesthesiologists as well as surgeons, with both professionals concentrating on their immediate role in the surgical procedure [23]. In addition, surgeons use prolonged SAP (7–10 days) in the belief that it may reduce the incidence of postoperative infections, including SSIs [7]. Non-adherence to guidelines is frequently motivated by fear of infection, i.e., prescribers perceive prolonged administration of (non-recommended) broader-spectrum drugs as safer [24,25,26,27]. Also, increased antibiotic use is often due to lack of local guidelines.

Overall compliance to SAP guidelines is usually in the range of 20–50% [28,29,30]. A review of SAP in the United Arab Emirates (performed on a diverse range of surgeries, 10.8% of which were orthopedic surgeries) found that antibacterial agent selection was concordant with the hospital guidelines in 25.7% of cases; this rate was found to be higher compared to Jordan (1.7%) and Iran (7.5%), but lower compared to India (68%) and the Netherlands (92%) [31]. In this review, guideline adherence in terms of agents was relatively high, 88.6% in the pre-intervention period, and increased to 90.1% in the intervention period. The use of unrecommended antibacterial agents remained almost the same, mostly due to prophylactic use of drug combinations (fluoroquinolones and amikacin, metronidazole, or rifampin) in revision arthroplasties where the risk for PJI is higher [32]. Nevertheless, the use of metronidazole in combinations decreased (by 2.8%, from 5% to 2.2%, *p* = 0.122) in the intervention period. Inappropriate use of metronidazole is not uncommon. A study in Pakistan on different surgical procedures, including orthopedic surgeries, found that after the pharmacist intervention the use of combination antibiotic therapies with metronidazole decreased significantly (from 26.2% to 16%, *p* = 0.011). However, in the same study the rate of inappropriate antibiotic choice did not change significantly, which is consistent with our findings [7]. Also, an Italian research group failed to show any significant improvement in the choice of antibiotics (78.4% vs. 78.4%, *p* = 0.48) [33].

In our study we found an overall guideline adherence (agent choice, dosage, timing, and duration) of 2% in the pre-intervention period. The adherence to guidelines was relatively high for agent choice (88.6%) and for dosage (86.3%); nevertheless, only 2% of these patients received SAP for an appropriate duration. Due to the shortening of SAP duration, the rate of overall guideline adherence (agent selection, dosage, and duration) improved significantly (increased by 56.2%, *p* ˂ 0.001) in the intervention period, which is in line with above-mentioned international findings.

During the intervention phase of the present study, slight improvement in the dosage of guideline adherent antibiotics (of 2.7%, from 86.3% to 89%, *p* > 0.05) was observed. This adherence rate for SAP dosage after pharmacist intervention is similar to rates of appropriateness of dosage reported in the literature. Zhou et al. reported an increase in appropriateness of dosage for cefuroxime (7.4%, from 77.0% to 84.4%, *p* = 0.01) after pharmacist intervention in SAP [11]. An Italian study in elective surgical procedures including orthopedic surgeries reported significant improvement after pharmacist intervention in correct dosage (increased by 14.1%, from 69.7% to 83.8%, *p* ˂ 0.001) [33].

As for timing, El Hassan et al. found a guideline adherence of 30.4% for the timing of the first dose [31]. Data regarding timing were not collected in this study. We believe that the practice of patients getting the first dose of prophylactic antibiotic on arrival in the operation room.

In the present study, SAP duration was observed to decrease significantly in the intervention period; improvement in SAP duration related adherence to the guidelines was recorded in 42.9% of the studied cases. The huge difference in the proportion of one-day prophylaxis in the two periods (59.8% increase, *p* < 0.001), as well as the reduction in the proportion of over five days prophylaxis to one-third of the original rate (11.6% decrease, *p* < 0.001) were particularly favourable changes (Table 2).

Several studies have focused on the duration of SAP. In Europe, data obtained in a point prevalence survey conducted by the ECDC (European Centre for Disease Prevention and Control) showed that antibiotic use for SAP accounted for 18% of the total antimicrobial use in acute care hospitals in the EU. In surgery, 36% of the administered antibiotics are used for SAP, and more than half (60%) of all these antibiotics were used for more than one day, which implies prolonged use. In Hungary, these rates are above the European average (45% and 56%, respectively) [34]. In a retrospective cohort study including 14,575 patients undergoing THA/TKA, 63.7% were administered prolonged SAP [35]. At the same time, in another retrospective observational study including 1019 patients with TKA/THA, only 21.7% of patients received prolonged SAP [36]. A recent retrospective study in Ethiopia including 188 patients found that 96% of patients received prolonged SAP (more than 24 h), and 61% of the patients were administered antibiotics for more than 72 h [37]. According to a clinical pharmacist’s study, in 60.5% of all orthopedic surgeries SAP was administered for a longer duration than 24 h [31]. After the pharmacist intervention in SAP (56.6% orthopedic surgeries), significant reductions were observed in the mean duration of antibiotic prophylaxis (17%, from 66.01 ± 41.015 to 55.20 ± 36.214 h, *p* = 0.003) [7]. Moreover, according to a randomized trial of 358 patients undergoing THA, TKA, or hip fracture repair, which was conducted to compare prophylaxis lasting 24 h to prophylaxis conducted for 7 days, no significant difference was found in SSI rates between groups [38].

### 3.2. Antibiotic Exposure and Cost

As our results show, pharmacist intervention can have a significant impact on SAP compliance resulting in a significant reduction in the amount of antibiotics used for SAP (by 41%, from 6.07 ± 0.05 to 3.58 ± 4.33 DDD/patient, *p* < 0.001) as well as in prophylactic cost (by 54.8%, *p* < 0.001), not only in primary arthroplasties, where the adherence to guidelines improved very much (56.2%, *p* < 0.001), but also in revision hip arthroplasties (by 50.5%, *p* > 0.05).

To our knowledge, there are no published data in the literature on the amount of antibiotic exposure in SAP in orthopedic wards. However, a study conducted at a surgical ICU with the aim to reduce SAP showed a significant reduction of cefuroxime use in SAP (14.4%, from an estimated mean of 1036 DDD/1000 to 887 DDD/1000 patient-days) after intervention [39]. A study on the role of clinical pharmacists in multidisciplinary teams found that the rate of SAP in clean wound surgery (THA, TKA) decreased by 80% in 5 years, entailing the decrease of the average cost of antibiotics per case (from 308.67 USD in 2009 to 69.75 USD in 2015) [40]. According to another study, pharmacist intervention in SAP reduced irrational use of antibiotics, leading to a decrease in the average total cost of antibiotic use (by 71.06% in USD) [20].

### 3.3. Clinical Outcomes

Regarding clinical outcomes, the most important limitation of our study is that data on hospital readmission due to SSIs may be incomplete due to the fact that only patients with severe SSI were readmitted to the hospital in both study periods (1.1% and 1%, respectively) (Figure 1), and we had no insight into non-severe cases. There may also be patients who were managed at some other hospital; these patients could not be covered by this study. Based on previous findings, SSIs are most likely to develop in the first 60 days; thus, most probably only a small proportion could have remained unreported in the present study [41,42].

We found a relatively low rate of confirmed SSIs for both periods (3% vs. 1.2%, *p* = 0.21). Beside this, median time to diagnosis of SSI in this study was eight days (ranging from 1 to 23 days). At the same time, we believe that these SSI rates are also confirmed by the fact that the number of cases requiring postoperative antibiotic treatment due to infections related to the surgery also decreased significantly (by 16.8%) (Table 2).

According to a literature review, significant variability in the incidence of orthopedic SSIs (due to perioperative circumstances, patients’ medical conditions) was noted between different studies (from 1.9% to 22.7%) [43,44]. A 4-year prospective cohort study performed in orthopedic patients in Belgrade found an overall SSI incidence rate of 2.8% (0.6% for THA, and 2.9% for TKA) [45]. A recent study found that the median time to diagnosis of SSI was 33 days (ranging from 1 to 355 days) [46]. Zhou et al. found that pharmacists’ interventions on SAP resulted in significantly decreased rates of SSIs (from 3.5% to 1.2%, *p* = 0.02) [11]. These SSI rates published by Zhou et al. before and after the intervention are almost identical to our findings.

The other clinical outcome we studied was the LOS. As the findings reported earlier reveal, neither prolonged prophylaxis, nor choosing agents with a broader spectrum than recommended, nor combinations provided any benefit in terms of length of stay [8,40,47,48]. At the same time, an experimental pre-post prospective study on orthopedic surgeries found that overall guideline adherent SAP (agent selection, dosage, and duration) was associated with a one-day decrease in LOS [49].

Some studies found direct association between pharmacist intervention and decrease of LOS; a study on different surgical procedures including orthopedic surgeries found that pharmacist intervention resulted in favorable outcomes with significantly decreased LOS (by 16.6%, from 5.4 ± 4.814 to 4.50 ± 3.398 days, *p* = 0.023) [7]. Xi et al. found that pharmacist intervention in SAP reduced significantly the average LOS (31.78%, from 17.64 ± 4.92 to 13.34 ± 2.05 days, *p* < 0.001) in patients undergoing hip arthroplasty [20]. Likewise, Zhou et al. observed that pharmacist intervention in SAP significantly reduced the average LOS (7.45%, from 23.3 ± 8.9 to 20.9 ± 8.9, *p* < 0.001). Our results support these findings by showing similar improvement in the intervention period, when LOS decreased significantly for all types of arthroplasties (by 37.2%, from 11.22 ± 6.96 to 7.62 ± 3.02 days, *p* < 0.001).

In the intervention period, the activity of the clinical pharmacist was not only welcome, but actively sought for. Studies also show that adherence to guidelines may be facilitated by consultation, and even more by regular audits of the prophylaxis practice [18,50].

### 3.4. Limitations and Strengths of This Study

One of the important limitations of our study is that this was a single-center study conducted in a university-affiliated hospital; therefore, findings may not be directly extrapolated to other settings. At the same time, this study provides detailed, first-hand observations of everyday work processes on SAP at an Orthopedic ward.

## 4. Materials and Methods

### 4.1. Study Design

This was a single center study including a retrospective observational part (pre-intervention period) and a prospective intervention part (intervention period). The study was conducted at a 61-bed Orthopedic Department of a tertiary-care center in Hungary. The pre-intervention period was from 1 November 2016 to 31 October 2017 while the intervention phase from 1 November 2017 to 31 May 2018 periods. The pre-intervention period was merely an observational period, when the clinical pharmacist was present in limited hours in the ward compared to the intervention period, and collected data retrospectively from all patients receiving an antibiotic. The intervention period was a prospective phase, when the clinical pharmacist spent six hours a day in the ward.

### 4.2. Study Population

The study population included all the hospitalized patients receiving SAP for primary THA (Total Hip Arthroplasty), TKA (Total Knee Arthroplasty), and revision arthroplasty (surgery performed to replace the worn-out joint) at the aforementioned Department.

### 4.3. Exclusion Criteria

All patients admitted from another ward/hospital who were on antibiotic treatment, patients receiving preoperative empirical antibacterial therapy due to various suspected or proven infections, or targeted antibacterial therapy, or SAP for other operations than THA and TKA, as well as patients readmitted due to SSI or PJI (primary TKA, THA, or revision arthroplasty) were excluded from the study.

### 4.4. Pre-Intervention and Intervention Period

The data obtained in the pre-intervention period were analyzed by the pharmacist, who later provided feedback to prescribers. Taking into account the HOA (Hungarian Orthopedic Association) national guideline and the clinical practice guidelines for SAP published by ASHP [8], problems related to the appropriate use of SAP (choice of agent, timing, dosing, and duration) were discussed.

Based on HOA and ASHP SAP guidelines, the recommended first-line systemic antibiotic for prophylaxis in arthroplasties was cefazolin or cefuroxime; for patients with allergy to beta-lactams clindamycin or vancomycin is recommended as an alternative; whereas in cases when the prevalence of Gram-negative pathogens is high, gentamicin or amikacin as an addition to cefazolin or cefuroxime [8]. Dose and duration of antibiotic use recommended for SAP are included in Table 3. In the intervention period, dose adjustment recommended by the clinical pharmacist was based on patient age, weight, and renal function. SAP was initiated and stopped by the surgical team based on the pharmacist’s recommendation. The clinical pharmacist’s interventions consisted of the following: proactively controlling the antibiotic therapy every day on an individual level to ensure compliance with SAP (agent, dosage, and duration) guidelines, attending afternoon surgical ward visits twice a week, and discussing their findings with the anesthesiologist and surgeons in cases when SAP guideline deviations were observed. Moreover, the pharmacist was involved in antibiotic related decisions and provided continuous counselling service continuously. The obtained data were compared to determine the results of the pharmacist led intervention.

Prophylaxis was considered appropriate if it did not last for more than one day for primary, and more than five days for revision arthroplasties.

### 4.5. Data Collection

All hospitalized patients receiving antibiotics for SAP were identified and their medical records were retrieved. Demographics (age, gender), clinical characteristics (weight, date of hospital admission and discharge, surgical diagnosis, date and type of surgery, LOS, antibiotic allergy, clinical signs of SSIs including redness, delayed healing, fever, pain, tenderness, warmth, swelling, and presence of pus produced by wound were observed on surgical ward round), symptoms: fever > 37.5 °C, C-reactive protein > 5 mg/L, positive blood culture or micro-organism isolated from wound, bone, or synovial fluid samples), data on antibacterial administration (indication, agents, dose, route of administration, frequency, duration of antibacterial treatment, antibiotic generic substitution/combination), and cost (cost of antibiotic agents) were recorded on data collection forms. LOS was calculated by subtracting day *of* admission from day *of* discharge, and refers to the number of days that patients spent in hospital. Both the admission and discharge day were counted as one day.

Patients were anonymized, and thus made unidentifiable in the study.

### 4.6. Data Analysis

We compared prophylactic antibiotic use regimen, namely the number of prescribed antibiotics (single agent or combination), active agent(s), dosage, and duration of prophylaxis, as well as and rate of guideline adherence, antibiotic exposure: DDD/patient, antibiotic costs, and clinical outcomes (LOS, number of surgical site infections) during the two study periods using chi-square, Fisher exact, and Mann-Whitney tests, as appropriate, using R statistical environment. To measure the antibiotic consumption, we applied the World Health Organization’s ATC/DDD index (version 2020). Defined Daily Dose (DDD) is the assumed average maintenance dose per day for a drug used for its main indication in adults. Antibiotic costs were calculated based on actual prices obtained from the central hospital pharmacy. Values of equal or less than 0.001 (multiple comparison method) were considered statistically significant.

## 5. Conclusions

Continuous presence of the clinical pharmacist in orthopedic surgery led to significant improvement of SAP guideline adherence and increased appropriate antibiotic use, which were accompanied by decrease in direct antibiotic cost, number of surgical site infections, and length of stay. Results suggest that clinical pharmacists acting as active members in antibiotic stewardship teams (providing consultation, monitoring of the administered antibiotics, and concomitant consultation with prescribers) may play an important role in promoting rational use of prophylactic antibiotics in surgery, and avoiding unnecessary SAP costs.

## Figures and Tables

**Table 1:** Basic and clinical patient’s characteristics in the pre- and intervention period.

Parameters	Pre-Intervention Period N = 395 (%)	Intervention Period N = 182 (%)	*p*-Values
Age (Mean ± SD)—years	65 ± 10.9	66 ± 10.5	0.292
18–65	174 (44.1%)	75 (41.2%)	0.685
65+	221 (55.9%)	107 (58.8%)	0.738
Gender			
Male	151 (38.2%)	70 (38.5%)	0.971
Female	244 (61.8%)	112 (61.5%)	0.979
Median body weight (range)-kg	59.9 (35–105)	62.1 (28–160)	0.675
Diagnosis primary arthroplasty			
Osteoarthritis	161 (40.8%)	73 (40.1%)	0.346
Osteonecrosis	195 (49.4%)	93 (51.1%)	0.823
Others *	16 (4%)	6 (3.3%)	0.671
Surgical procedure			
THA	250 (63.3%)	93 (51.1%)	0.156
TKA	122 (30.9%)	79 (43.4%)	0.044
Revision hip arthroplasty	19 (4.8%)	7 (3.8%)	0.619
Revision knee arthroplasty	4 (1%)	3 (1.7%)	0.522

SD: standard deviation, THA-Total Hip Arthroplasty, TKA-Total Knee Arthroplasty, * Others: Avascular Necrosis, Fracture Neck of Femur, Ankylosing Spondylitis, Rheumatoid Arthritis.

**Table 2:** Impact of pharmacist intervention on surgical antibacterial prophylaxis (SAP).

Parameters	Pre-Intervention Period N = 395 (%)	Intervention Period N = 182 (%)	Increase/ Decrease %	*p*-Values
Number of antibiotics used simultaneously: 1	371 (93.9%)	173 (95.1%)	1.2%	0.925
Number of antibiotics used simultaneously: 2–3	24 (6.1%)	9 (4.9%)	−1.2%	0.607
Guideline adherent antibiotic	350 (88.6%)	164 (90.1%)	1.5%	0.897
Cefuroxime	348 (88.1%)	160 (87.9%)	−0.2%	0.987
Cefuroxime + amikacin	2 (0.5%)	4 (2.2%)	1.7%	0.066
Guideline non-adherent antibiotic	45 (11.4%)	18 (9.9%)	−1.5%	0.629
Co-amoxiclav	1 (0.3%)	-	−0.3%	0.497
Ciprofloxacin	22 (5.6%)	13 (7.1%)	1.5%	0.490
beta-lactams or FQ + metronidazole	20 (5%)	4 (2.2%)	−2.8%	0.122
beta-lactams or FQ + rifampin	1 (0.3%)	-	−0.3%	0.497
FQ + amikacin	1 (0.3%)	1 (0.6%)	0.3%	0.575
Guideline-adherent agent(s)	350 (88.6%)	164 (90.1%)	1.5%	0.897
Guideline-adherent agent, dosage	341 (86.3%)	162 (89%)	2.7%	0.815
Guideline-adherent agent, dosage, and duration	8 (2%)	106 (58.2%)	56.2%	˂0.001
Duration of prophylaxis—days (Mean ± SD/Median)	4.08 ± 2.08 (3)	2.42 ± 1.90 (1)	−42.9%	˂0.001
One day prophylaxis	9 (2.3%)	113 (62.1%)	59.8%	˂0.001
Three days prophylaxis	135 (34.2%)	20 (11%)	−33.2%	˂0.001
Over five days prophylaxis	72 (18.2%)	12 (6.6%)	−11.6%	˂0.001
Guideline adherent duration	20 (5%)	117 (64.3%)	59.3%	˂0.001
Primary arthroplasties	8 (2%)	111 (61%)	59%	˂0.001
Revision arthroplasties	12 (3%)	6 (3.3%)	0.3%	0.872
DDD/patient (Mean ± SD)	6.07 ± 0.05	3.58 ± 4.33	−41%	˂0.001
LOS—days (Mean ± SD/Median)	11.22 ± 6.96 (9)	7.62 ± 3.02 (7)	−37.2%	˂0.001
SSIs onset—days (Mean ± SD/Median)	8.91 ± 5.75 (8)	8.5 ± 6.61 (8)	−3%	0.170
Suspected SSIs	43 (11.6%)	4 (2.3%)	−9.3%	˂0.001
Confirmed SSIs	11 (3%)	2 (1.2%)	−1.8%	0.214
Need for postoperative antibiotic treatment due to SSIs	77 (19.5%)	5 (2.7%)	−16.8%	˂0.001
Prophylactic antibiotic cost/patient—HUF (Mean ± SD)	9278.79 ± 6094.29	3598.16 ± 3354.55	−54.8%	˂0.001
Primary Arthroplasties	8768.70 ± 4478.91	3162.23 ± 2641.7	−56.2%	˂0.001
Revision Arthroplasties	17,528.96 ± 15,852.28	9793.4 ± 6732.08	−50.5%	0.070

DDD—Daily Defined Dose; beta-lactams: cefuroxime, co-amoxiclav; FQ: ciprofloxacin; SD: standard deviation; LOS—Length of Stay; SSIs—Surgical Site Infections.

**Table 3:** Dose and duration of antibiotic use recommended for SAP.

Active Agent	Dose (Adults)	Duration	
		In Primary Arthroplasties	In Revision Arthroplasties
cefazolin	1–2 g iv q8h	up to 24 h	up to 5 days
cefuroxime	1.5 g/750 mg iv q8h		
clindamycin	600/400 mg iv q8h		
vancomycin	500 mg iv q6h		
	1 gmg iv q12h		
amikacin	15 mg/kg/day iv q8–12h		
gentamicin	3–6 mg/kg/day iv q12–24h		

## Data Availability

Data are available from the corresponding author upon reasonable request.
